# Post-Exercise Appetite and Ad Libitum Energy Intake in Response to High-Intensity Interval Training versus Moderate- or Vigorous-Intensity Continuous Training among Physically Inactive Middle-Aged Adults

**DOI:** 10.3390/nu10101408

**Published:** 2018-10-02

**Authors:** Eric Tsz-Chun Poon, Feng-Hua Sun, Anthony Pui-Wan Chung, Stephen Heung-Sang Wong

**Affiliations:** 1 Department of Sports Science and Physical Education, The Chinese University of Hong Kong, Hong Kong, China; ericpoontc@link.cuhk.edu.hk (E.T.-C.P.); a-chung92@hotmail.com (A.P.-W.C.); 2 Department of Health and Physical Education, The Education University of Hong Kong, Hong Kong, China; fhsun@eduhk.hk

**Keywords:** high-intensity interval training, interval training, energy intake, appetite responses, weight management, public health

## Abstract

High-intensity interval training (HIIT) is considered a time-efficient exercise strategy for weight management. However, data regarding the acute appetite and energy intake responses to HIIT versus continuous training remain inconclusive. This study investigated the ad libitum energy intake and appetite responses to a single session of HIIT versus moderate-intensity continuous training (MICT) and vigorous-intensity continuous training (VICT). Using a randomized crossover design, 11 middle-aged physically inactive men (45.7 ± 7.4 years, 23.5 ± 2.1 kg m^−2^) participated in three treadmill trials at 7-day intervals. HIIT comprised 10 1-min periods at 100% VO_2max_ interspersed with 1-min periods of active recovery. MICT comprised a 40-min session at 65% VO_2max_, while VICT comprised a 20-min session at 80% VO_2max_. After each trial, the participants consumed an ad libitum buffet meal for which the energy intake was recorded. The participants’ perceived appetite was assessed before and after exercise sessions using the Visual Analogue Scale (VAS). No significant differences in post-exercise ad libitum energy intake were observed between trials (HIIT: 645 ± 262.9 kcal; MICT: 614.7 ± 271.2 kcal; VICT: 623.1 ± 249.0 kcal, *p* > 0.05). Although the perceived appetite responses exhibited a significant main effect of time (*p* < 0.01), no group differences were observed (*p* > 0.05). In summary, these findings suggest that the interval or continuous nature of exercise has no significant effect on appetite responses in physically inactive middle-aged adults, at least during the short-term post-exercise period.

## 1. Introduction

Physical inactivity, which increases the risks of obesity and associated cardio-metabolic diseases, has been identified as a global pandemic [1]. By contrast, exercise can profoundly affect weight loss by increasing energy expenditure and subsequently creating a negative energy balance [2]. However, the effects of exercise on acute appetite responses and subsequent food intake remain controversial. Relevant previous studies focused mainly on continuous training for periods of 30–120 min and at intensities of 36–81% VO_2max_ [3]. Additionally, evidence suggests that exercise may trigger changes in the levels of circulating appetite-related hormones and/or metabolites and sensations of hunger and satiety [4]. These responses also appear to depend on exercise intensity [5], as an increased intensity level was shown to promote appetite suppression [6]. However, prolonged, continuous low-to-moderate exercise may not be practically feasible in some individuals who perceive a lack of time as a barrier to exercise [7].

Recent public health promotions have highlighted high-intensity interval training (HIIT) as a time-efficient exercise strategy [8]. This method, which has historically been used by athletes as a training method, has recently attracted more widespread popularity among the general population and was ranked first in the American College of Sports Medicine (ACSM) Worldwide Survey of Fitness Trends for 2018 [9]. A HIIT workout typically involves repeated bouts of high-intensity exercise (usually ≥80% of the maximum heart rate), interspersed with active or inactive periods of recovery [10,11]. Some research suggests that despite the substantially reduced training volume and lower time commitment relative to a traditional continuous training approach, HIIT not only induces favorable changes in body composition [12,13] but also suppresses the post-exercise appetite [14]. Nevertheless, only a few previous studies have directly compared acute energy intake and appetite responses after HIIT versus continuous training, and these have yielded equivocal findings [15,16,17]. Other studies have investigated supra-maximal or ‘all-out’ HIIT protocols, which are performed at a VO_2max_ of >100% [15] but may be considered too aversive for relatively inactive populations [16]. Therefore, it remains unclear whether a practical model of HIIT that most individuals (especially middle-aged adults with relatively sedentary lifestyles) are more likely to perform in a real-world environment would elicit similar appetite and energy intake responses as a typical, long-duration moderate-intensity continuous training (MICT) program. Furthermore, it remains unknown whether the interval nature of HIIT would affect appetite responses when compared to a time and energy-matched vigorous-intensity continuous training (VICT) protocol.

Elucidation of the relationships among exercise, appetite and diet would be valuable to the designs of effective exercise training programs that target weight management in healthy, subclinical and even clinical populations. Therefore, this study aimed to compare the ad libitum energy intake and appetite responses after a single session of HIIT versus MICT and VICT in physically inactive middle-aged men. We hypothesized that the post-exercise ad libitum energy intake and perceived appetite would not differ between all trials.

## 2. Materials and Methods

### 2.1. Participants

**Figure 1:**
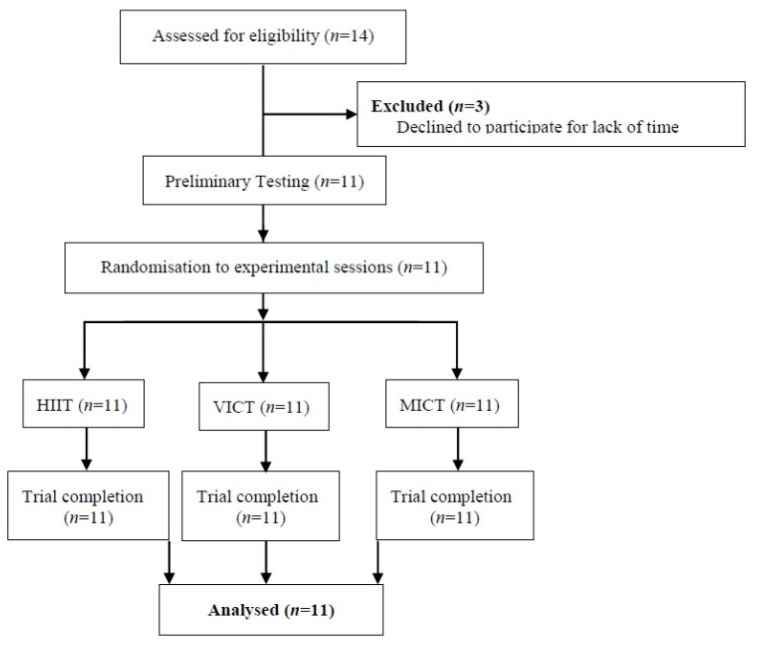
Selection flowchart of participants. HIIT: High-intensity interval training; MICT: Moderate-intensity continuous training; VICT: Vigorous-intensity continuous training.

Eleven healthy, physically inactive middle-aged men (age range: 40–59 years) were recruited to participate in the study via advertisements placed at the authors’ university, partner institutions, community centers and online (see Figure 1 for the selection flow and Table 1 for the demographic characteristics of the participants). Physical inactivity was defined as performing fewer than 150 min of moderate or 75 min of vigorous physical activity per week for a period of ≥3 months, as suggested by current physical activity guidelines [17]. Eligible participants included those who had not participated in any structured exercise training and dieting program during the past year. The exclusion criteria included severe high blood pressure (≥180/100 mmHg), use of medication for chronic disease and myocardial infarction, uncompensated heart failure or unstable angina pectoris during the previous 4 weeks, as suggested by current exercise prescription guidelines [18]. All eligible participants were also screened by a certified Exercise Physiologist to confirm absence of high cardiovascular risks using a health history questionnaire [18].

The sample size required for the study, 11 participants, was based on an anticipated small effect size (i.e., ES = 0.4) of the energy intake responses; this estimation had an α  value of 0.05 and β  value of 0 .20 (G*Power version 3.0.10) and reflected related research [19,20]. Detailed explanations of the aim, procedure, benefits and potential risks of the study were given to participants and all participants subsequently provided written informed consent. The study was conducted in accordance with the Declaration of Helsinki and procedures were submitted to and approved by The Ethics Committee at The Chinese University of Hong Kong (CREC Ref. 2017-.057-T).

### 2.2. Preliminary Testing

During the first laboratory visit, the participants’ heights were measured using a stadiometer (Seca, Leicester, UK). The participants’ body weights, body mass indices (BMI) and body fat percentages were determined using a body composition analyzer (MC-780MA, Tanita Corp., Tokyo, Japan). VO_2max_ values were determined during a continuous, incremental, graded uphill treadmill running test to volitional exhaustion, based on a protocol reported previously by our laboratory [21]. VO_2max_ was determined using the following standardized criteria: (1) a respiratory exchange ratio of ≥1.10 and (2) failure of the heart rate (HR) to increase with an increasing workload [18]. The HR was recorded continuously during the test using HR telemetry (H10 Sensor, Polar Electro, Kempele, Finland). The running intensities (as % VO_2max_) prescribed for the subsequent experimental trials were based on the corresponding velocity attained during the VO_2max_ test.

### 2.3. Familiarisation Trial

The second visit to the laboratory involved a familiarization trial, which was completed 1 week after the VO_2max_ test. This trial was intended to familiarize the participants with the experimental procedures and to confirm whether the individually prescribed running intensity met the designated percentage VO_2max_ threshold of each protocol [22,23]. In this trial, the participants were required to complete half of each protocol used in the main trials, with a 30-min rest between protocols. The protocol sequence was randomized during the familiarization trial.

### 2.4. Experimental Trials

One week after the familiarization trial, the participants completed one of 3 experimental trials on a standardised treadmill (Pulsar 3p, h/p/cosmos sports and medical, GmbH, Nußdorf, Germany) in a randomized and crossover order. The HIIT protocol comprised 10 1-min bouts running at 100% VO_2max_, separated by 1-min intervals of active recovery at 50% VO_2max_. The total exercise duration was 20 min. The MICT protocol comprised 40 min of running at 65% VO_2max_. The VICT protocol comprised 20 min of running at 80% VO_2max_. The energy expenditure (EE) was estimated via indirect calorimetry (Max-II metabolic cart system, AEI Technologies, Pittsburgh, PA, USA) with the assumption of a non-protein respiratory exchange ratio [24]. Based on our laboratory pilot data, we attempted to match the EEs of HIIT and VICT (but not of MICT) and this matching was confirmed upon further analysis (see Section 3.3). Notably, the current methodological approach was adapted from several previous studies [25,26] with the intent to provide greater real-life implications, and reflected the relatively low-volume and time-efficient nature of HIIT and VICT, compared with the traditional high-volume and long-duration nature of MICT. HR telemetry was used for continuous HR monitoring throughout the tests, as described above.

All of the participants performed a 2-min warm-up at 50% VO_2max_ and a 2-min cool-down at a self-selected light intensity (determined during the first trial and kept constant during subsequent trials). No external stimuli (e.g., music, television and mobile devices) or verbal encouragement was provided during the trials. The three trials were performed at 1-week intervals. For all trials, the participants arrived at the laboratory at the same time of day (between 8:00 and 11:00 a.m.) to eliminate any circadian effects. A standardised breakfast comprising wholegrain cereal (Weetabix Original, Burton Latimer, UK) and sport powder (Herbalife 24 Formula 1, Los Angeles, CA, USA) was provided to each participant at a carbohydrate (CHO) dose of 1.5 g/kg body mass, in accordance with a previous study [27]. After breakfast, the participants rested in a quiet room for 1 h before the commencement of exercise. 

### 2.5. Post-Exercise Energy Intake

After exercise, the participants again rested for 1 h in a quiet room. Subsequently, they were provided with a buffet meal for a fixed duration of 30 min and instructed to eat ad libitum until they achieved a self-regulated satisfactory level of satiety (i.e., ‘comfortably full’). The items available at the buffet were purchased from a local supermarket and varied with respect to CHO, fat and protein contents. The items included white and whole-wheat bread, three varieties of cereal, butter, margarine, ham, cheese, plain and fruit yoghurt, granola bars, chocolates, biscuits, apple juice, full-fat and skimmed milk and soft and sports drinks. Every food choice was weighed before and after consumption with a digital scale (KD-321, Tanita Corp., Tokyo, Japan) and recorded on a food log sheet for analysis. The nutrient contents of the consumed food were analyzed based on the manufacturers’ values and performed with food analysis software (The Food Processor^®^ version 11.4.412, ESHA Research, Salem, MA, USA). The following actions were taken to minimize any environmental and social factors that may influence eating behavior: (i) the participants always used the same plates and utensils, (ii) the research staff left the buffet room during consumption, (iii) the buffet foods were presented identically during each trial and (iv) food was presented in excess of the expected consumption, with additional food available upon request.

### 2.6. Perception of Appetite

The validated 100-mm Visual Analogue Scale (VAS) [28] was used to assess the subjective appetite at 3 time points: (i) immediately prior to exercise; (ii) immediately after exercise and (iii) 1-h post-exercise (before the buffet meal). This scale comprised four questions anchored with words representing the opposing extreme states of desire to eat, hunger, fullness and prospective food consumption at either end, as shown below.

(1) How strong is your desire to eat? (‘very weak’ to ‘very strong’),
(2) How hungry do you feel? (‘not hungry’ to ‘as hungry as I’ve ever felt’),
(3) How full do you feel? (‘not full at all’ to ‘very full’), and
(4) How much food do you think you could eat? (‘nothing at all’ to ‘a large amount’).

The average appetite score was calculated using the following formula:

Appetite score = (desire to eat + hunger + (100-fullness) + prospective consumption)/4

### 2.7. Blood Lactate

Blood lactate concentrations were recorded immediately before and after exercise (i.e., before cool-down) during all trials. Capillary blood samples (approximately 25 μL) were acquired from the fingertips using a portable analyser (Lactate Plus, Nova Biomedical, Waltham, MA, USA).

### 2.8. Dietary and Exercise Training Control

The participants were requested to avoid strenuous exercise, caffeine and alcohol for 24 h before all experimental trials. They were also asked to report their food intake during the previous 24 h at the first trial and to consume the same foods on the days before all subsequent trials.

### 2.9. Statistical Analysis

Data are presented as means ± standard deviations (SDs). SPSS for Windows (Version 20; IBM, Armonk, NY, USA) was used for the analysis. A series of two-way analyses of covariance (ANCOVA) with baseline measure as a covariate was used to determine the main effects of the exercise mode and time and their respective interactions with the perception of appetite and blood lactate concentration. Additionally, a series of one-way ANOVAs with repeated measures followed by Bonferroni’s post hoc test was conducted to examine the differences in post-exercise energy intake and macronutrients. The significance level (*p*-value) was set at 0.05.

## 3. Results

All participants completed the experiment successfully, and no adverse events were reported. No differences at baseline levels were observed in any measurements during the three trials.

### 3.1. Post-Exercise Energy Intake

No significant differences in post-exercise ad libitum energy intake and macronutrients were observed between the three trials (Table 2, all *p* > 0.05).

### 3.2. Perception of Appetite

**Figure 2:**
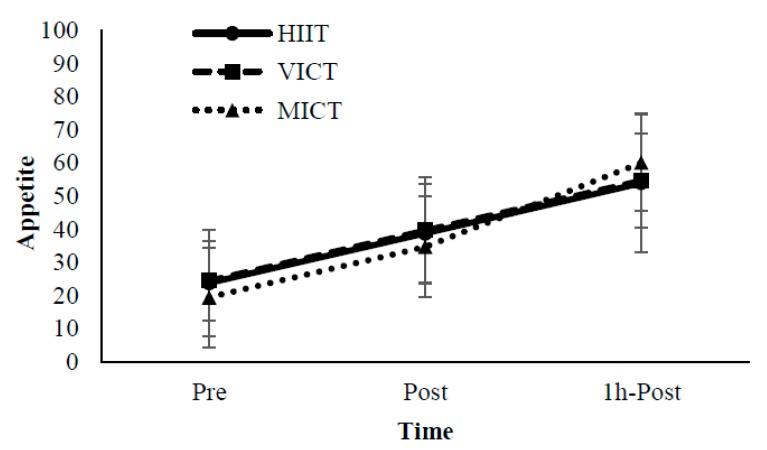
Appetite Scores. Pre: Immediately pre-exercise; Post: Immediately post-exercise; 1h-Post: 1-h post-exercise.

The perceived appetite scores are shown in Figure 2. A significant main effect of time was observed (*p* < 0.01). However, no significant main effects of protocols and their interactions were observed (*p* > 0.05).

**Figure 3:**
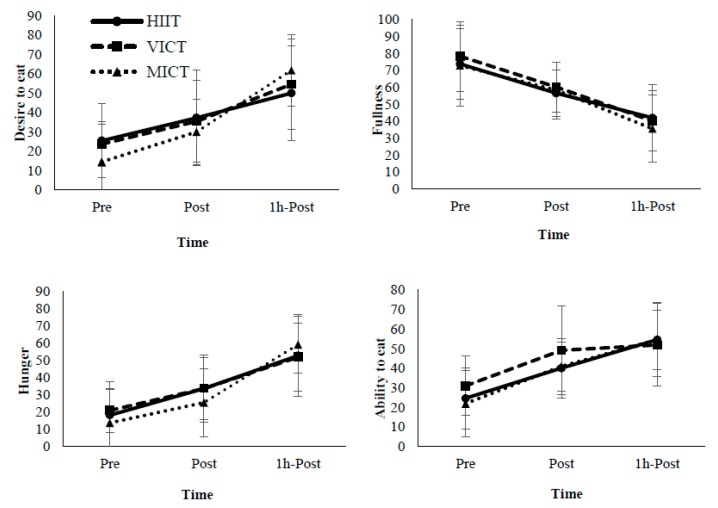
Appetite Sub-Score Analysis. Pre: Immediately pre-exercise; Post: Immediately post-exercise; 1h-Post: 1-h post-exercise.

As previously described, the appetite score comprised four sub-scores: desire to eat, hunger, fullness and ability to eat. In a further analysis (Figure 3), all sub-scores exhibited a significant main effect of time (*p* < 0.01) but no significant main effects of protocols and their interactions (*p* > 0.05).

### 3.3. Energy Expenditure and Blood Lactate

**Figure 4:**
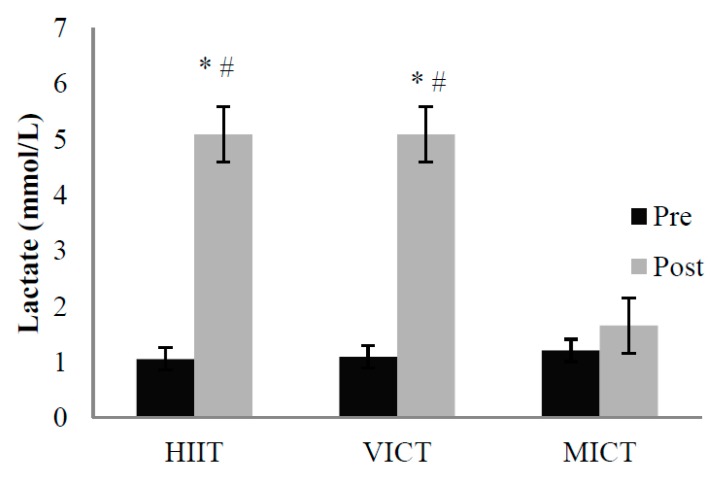
Changes in Blood Lactate Concentrations. * *p* < 0.01 versus pre-exercise; # *p* < 0.01 versus MICT.

The total energy expenditure (EE) during exercise was significantly higher during MICT (365 ± 43 kcal) than during HIIT (216 ± 29 kcal) and VICT (222 ± 32 kcal) (both *p* < 0.01). The EEs during HIIT and VICT were equivalent (*p* > 0.05). The blood lactate concentration was significantly higher immediately after HIIT (5.1 ± 0.5 mmol/L) and VICT (5.1 ± 0.6 mmol/L) than after MICT (1.7 ± 0.3 mmol/L) (both *p* < 0.05) (Figure 4).

## 4. Discussion

In the present study, the most notable finding was the lack of differences in the post-exercise ad libitum energy intakes and perceived appetites of physically inactive middle-aged adults in response to an acute bout of HIIT versus MICT or VICT. These findings suggest that short-duration vigorous exercise, regardless of the interval or continuous nature, yielded similar appetite responses as conventional, higher volume MICT, at least during the short-term post-exercise period.

The physiological benefits of HIIT on weight loss and metabolic health have been reviewed extensively in recent years [10,13,29,30,31]. Apart from direct energy consumption during the workout, changes in acute appetite responses have also been proposed as a benefit of HIIT on fat loss [30]. However, studies of the effects of HIIT on acute appetite perceptions and post-exercise ad libitum energy intake have yielded conflicting findings. Some studies reported comparable effects of HIIT and MICT on both subjective appetite perceptions and ad libitum energy intake [32,33,34]. For instance, one study [33] that compared the effects of isocaloric (250 kcal) bouts of MICT and HIIT or short-duration HIIT (125 kcal) in 12 overweight/obese individuals observed similar subjective appetite sensations and post-exercise energy intakes. Likewise, in another study [34] of the effects of MICT (50-min at 70% VO_2max_) and HIIT (6 × 3-min at 90% VO_2max_ with 3-min active recovery) in combination with short exposure to hypoxia on appetite in 12 healthy male participants, the appetite responses did not appear to be influenced by the exercise modality. A more recent study [35] of 12 obese men also suggested that neither HIIT (10 × 1-min at 90% HR_max_ with 1-min recovery) nor MICT (20-min at 70% HR_max_) altered the energy intake at 1 or 24 h post-exercise, despite an apparent transient reduction in hunger immediately following HIIT.

Nevertheless, some studies reported significant differences when more intense HIIT protocols were adopted, such as those with supra-maximal intensities (>100% VO_2max_) or an ‘all-out’ sprint interval training (SIT). One study [15] examined the comparative appetite and energy balance responses between SIT (6 × 30-s sprints) and traditional MICT (60-min at 65% VO_2max_) in 12 healthy men. Notably, appetite was more acutely suppressed during SIT than during MICT, although the ad libitum energy intake did not differ between trials. Sim et al. (2014) recruited 17 overweight male adults to complete four crossover 30-min trials, including resting control, MICT (65% VO_2max_), HIIT (alternating 60 s at 100% VO_2max_ and 240 s at 50% VO_2max_) and supra-maximal HIIT (alternating 15 s at 170% VO_2max_ and 60 s at 50% VO_2max_), followed by an isocaloric meal after each trial and an ad libitum meal 70 min later. No significant differences in perceived appetite were observed between the trials of that study, consistent with the present study findings. However, the ad libitum energy intake was lower after the supra-maximal HIIT trial than after the MICT trial. Based on this observation, the authors concluded that supra-maximal HIIT may suppress subsequent ad libitum energy intake in overweight inactive males. Although this finding appears to contradict our current observation of a lack of difference in post-exercise energy intakes between trials, it may be explained by the fact that the supra-maximal HIIT protocol in the study by Sim and colleagues utilized a much higher intensity (i.e., 170% VO_2max_) than the current HIIT protocol (i.e., 100% VO_2max_). Hence, a certain level of HIIT intensity appears to be required to suppress the actual food intake and appetite response. However, the practicality of supra-maximal HIIT and SIT for the general population has been consistently challenged [16,36]. In addition, it is worth noting that the energy expenditures of HIIT and MICT in the study by Sim and other related research [20] were matched. This methodological design may have limited the practical implication, as it could not encapsulate the low-volume and time-efficient nature of HIIT as generally performed by the public.

Our analysis also revealed higher blood lactate concentrations immediately after HIIT and VICT (both *p* > 0.05) than after MICT. Lactate is a metabolite that has been suggested as a key suppressor of energy intake [31,37]. In particular, the anorexigenic effect of lactate has been observed in healthy adults under euglycemic conditions and may be due to the interaction of this molecule with glucose sensors in the central nervous system [38]. Despite this significant difference in lactate concentrations, however, the appetite responses were similar among the trials. Accordingly, the regulation of another neuroendocrine system might be dominant at the physiological level [5,39]. We further speculated that the differences in exercise duration or energy expenditure between the trials in this study might have offset some of the effect of exercise intensity on appetite. The present study suggests that the actual difference in appetite might be trivial when comparing lower-volume vigorous exercise (either interval or continuous) with typical, longer-duration MICT. However, further work is needed to elucidate in detail how these factors interact to influence short-term appetite responses and post-exercise energy intakes. In addition, individual variability in food intake responses should not be overlooked, as the present data revealed a relatively large variance in total energy intake. Moreover, the potential effects of obesity level and sex on the appetite response to HIIT remains under debate [35,40]. Accordingly, future studies comparing normal-weight and obese individuals of different sexes are also warranted.

This study had several strengths, including the use of a within-subject, randomized cross-over design to investigate appetites and post-exercise energy intakes in response to HIIT versus both MICT and VICT. In addition, the protocol design used in the current study encapsulated the low-volume and time-efficient nature of HIIT when compared with longer-duration MICT and thus should provide more real-life implications. Despite these strengths, one major limitation of the present study was the lack of direct measurements of appetite-related hormones, such as ghrelin, peptide tyrosine-tyrosine, glucagon-like peptide-1 and pancreatic polypeptide, which hinders further interpretation from a mechanistic perspective. Nevertheless, the potential roles of these hormones on appetite-regulating mechanisms have been outlined in a recent review paper [5]. We do believe, however, that our findings provide valuable insights regarding the actual food intakes and appetite responses after an acute bout of HIIT versus both MICT and VICT. From a practical point of view, the similar effects observed among trials may suggest that both low-volume vigorous exercise (either interval or continuous) or higher volume MICT could be considered by healthcare professionals when designing programs that target weight management in physically inactive adults.

## 5. Conclusions

In summary, our findings did not reveal distinct differences in the post-exercise ad libitum energy intake and perceived appetite after an acute bout of HIIT versus MICT or VICT in a cohort of physically inactive middle-aged adults. These findings suggest that the appetite responses following short-duration vigorous exercise (either interval or continuous) do not differ from those following conventional, higher volume MICT, at least during the immediate post-exercise period. Future studies in normal-weight and obese individuals of different sexes are warranted to further elucidate the food intake and appetite relationships with different exercise modalities.

## Figures and Tables

**Table 1:** Demographic characteristics of the participants.

Variable	(*n* = 11)
Age (years)	45.7 ± 7.4
Height (cm)	168.4 ± 6.8
Weight (kg)	66.6 ± 8.3
Body fat (%)	20.1 ± 3.5
BMI (kg m^−2^)	23.5 ± 2.1
VO_2max_ (mL min^−1^ kg^−1^)	38.6 ± 5.4

Values presented in mean ± SD. BMI: Body mass index; VO_2max_: Maximal oxygen uptake.

**Table 2:** Energy intake and macronutrients during ad libitum buffet meals.

	HIIT	VICT	MICT
Energy Intake (kcal)	645 ± 262.9	623.1 ± 249.0	614.7 ± 271.2
Carbohydrates (g)	102.6 ± 41.8	96.9 ± 40.2	92.8 ± 40.3
Protein (g)	24.8 ± 9.9	26.3 ± 12.8	23.0 ± 10.2
Fat (g)	17.5 ± 6.8	17.2 ± 7.6	17.2 ± 8.6

Values presented in mean ± SD. HIIT: High-intensity interval training; MICT: Moderate-intensity continuous training; VICT: Vigorous-intensity continuous training.
